# Inhaled anesthesia associated with reduced mortality in patients with stage III breast cancer: A population-based study

**DOI:** 10.1371/journal.pone.0289519

**Published:** 2024-03-01

**Authors:** Emily Tzu-Jung Kuo, Chin Kuo, Cheng-Li Lin

**Affiliations:** 1 Department of Anesthesiology, China Medical University Hospital, Taichung, Taiwan (R.O.C.); 2 College of Artificial Intelligence, National Yang-Ming Chiao Tung University, Tainan, Taiwan (R.O.C.); 3 School of Medicine, Duke University, Durham, NC, United States of America; 4 College of Medicine, China Medical University, Taichung, Taiwan (R.O.C.); 5 Management Office for Health Data, China Medical University Hospital, Taichung, Taiwan (R.O.C); Umass Chan Medical School, UNITED STATES

## Abstract

**Background:**

Patients diagnosed with stage III breast cancer often undergo surgery, radiation therapy, and chemotherapy as part of their treatment. The choice of anesthesia technique during surgery has been a subject of interest due to its potential association with immune changes and prognosis. In this study, we aimed to compare the mortality rates between stage III breast cancer patients undergoing surgery with propofol-based intravenous general anesthesia and those receiving inhaled anesthetics.

**Methods:**

Using data from Taiwan’s National Health Insurance Research Database and Taiwan Cancer Registry, we identified a cohort of 10,896 stage III breast cancer patients. Among them, 1,506 received propofol-based intravenous anesthetic maintenance, while 9,390 received inhaled anesthetic maintenance. To ensure comparability between the two groups, we performed propensity-score matching.

**Results:**

Our findings revealed a significantly lower mortality rate in patients who received inhaled anesthetics compared to those who received propofol-based intravenous anesthesia. Sensitivity analysis further confirmed the robustness of our results.

**Conclusions:**

This study suggests that inhaled anesthesia technique is associated with a lower mortality rate in clinical stage III breast cancer. Further research is needed to validate and expand upon these results.

## Introduction

Breast cancer is the most common diagnosed cancer globally and the second-leading cause of cancer-related death in the United States. Data sourced from The Global Cancer Observatory, an initiative focused on collating and disseminating comprehensive cancer research, reveals a global cumulative incidence rate of 5.20% for breast cancer [1]. Distressingly, an estimated 685,000 women lost their lives to breast cancer in 2020 [2]. Most patients received surgical interventions, making it crucial to explore the impact of perioperative factors on patient prognosis. Consequently, growing interests in understanding the role of perioperative factors in treatment outcomes and long-term survival for breast cancer patients.

The choice of anesthetic agents can impact both the host immune response and the progression of minimal residual disease in breast cancer. Despite the optimal treatment, minimal residual disease, characterized by circulating tumor cells (CTCs) and disseminated tumor cells (DTCs), continues to pose significant challenges due to subsequent local relapse and distant metastasis [3]. Generally, studies conducted on inhaled anesthetics have revealed their immunosuppressive and pro-inflammatory effects, as well as the potential to promote angiogenesis and cellular proliferation, facilitating the spread of cancer cells in various in vivo, in vitro, and animal models [4, 5]. On the other hand, propofol, commonly used in total intravenous anesthesia (TIVA), has been suggested to possess anti-inflammatory, antioxidative, and antitumor properties by directly regulating key pathways and signaling in cancer cells [6, 7]. However, the existing literature yields inconsistent findings, with some recent studies proposing a protective role for volatile agents [8]. Consequently, the effects of anesthetic agents on the progression of breast cancer remain incompletely understood, and conflicting evidence persists in preclinical research.

**Fig 1:**
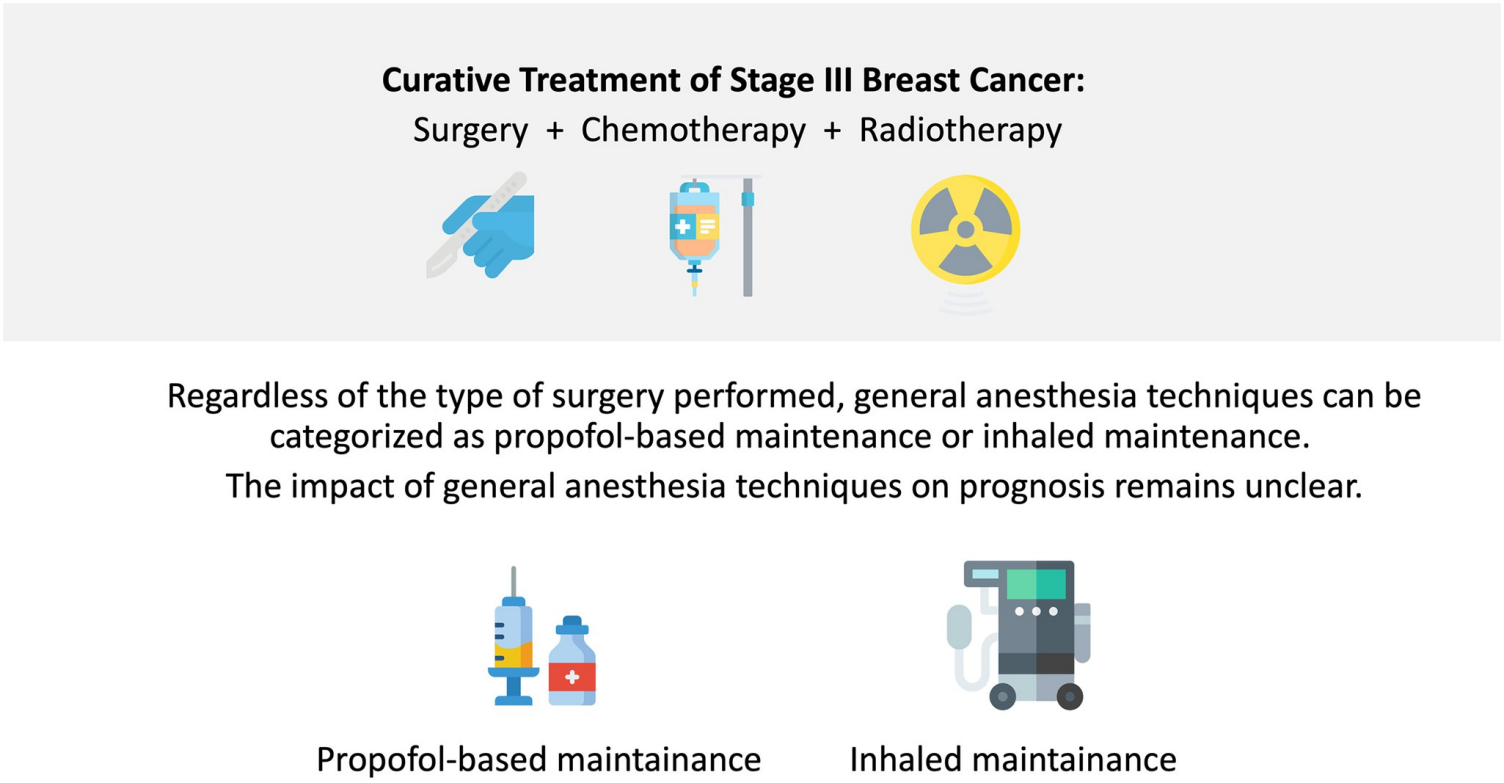
Topic overview. Anesthesia techniques for curative surgery in stage III breast cancer can be categorized into two types: propofol-based maintenance or inhaled agent maintenance. This article aims to explore and compare the outcomes associated with these two techniques. Icon made by Freepik from www.flaticon.com.

Breast cancer tumors were traditionally considered as “immune quiescence,” with limited lymphocyte infiltration, low mutational burden, and modest response rates to anti-PD-1/PD-L1 monotherapy [9]. However, recent tumor and immunologic profiling has revealed potential mechanisms of immune evasion in breast cancer and unique aspects of the tumor microenvironment (TME) [9–12]. Crosstalk within the TME involving the extracellular matrix (ECM), vasculature, stromal cells, immune cells, and endothelial cells undergoes changes as the tumor progresses or in response to specific treatments [12]. Studies have demonstrated alterations in host immunity, including dysfunction and decreased numbers of Natural Killer (NK) cells associated with clinical stage [13, 14]. Additionally, adaptive immunity responds differently in advanced stages, with increased numbers of Regulatory T cells (Tregs) observed in the peripheral blood of breast cancer patients, correlating with invasive breast cancer [15, 16]. Given the differential host immune responses between early and advanced stages, we hypothesize that the impact of inhaled anesthetics and propofol-based intravenous anesthesia would vary in locally advanced breast cancer. However, the majority of the clinical studies have predominantly focused on early-stage breast cancer, specifically stage I and II [17–19]. Thus, this study aims to investigate potential differences in mortality rates between stage III breast cancer patients who undergo surgery with propofol-based intravenous general anesthesia and those who receive inhaled anesthesia. The overview of the topic discussed in this article is presented in Fig 1.

## Materials and methods

### Database

Data were collected from Taiwan’s National Health Insurance Research Database (NHIRD) and linked with the Taiwan Cancer Registry (TCR). Taiwan has a single-payer healthcare system known as the National Health Insurance (NHI) Program, which has provided health insurance coverage to over 99.9% of the population since 1995 [20]. The NHIRD, derived from the NHI program, contains detailed information on medical claims, orders, expenses, prescriptions, and diagnoses for both inpatient and outpatient care. The Taiwan Cancer Registry, established in 1979, has high data quality and registered completeness of up to 97% [21]. We conducted a retrospective cohort study based on the population using de-identified data and accessed the database between August 15, 2021, and January 31, 2022, for research purposes. Ethical approval for the study was obtained from the Research Ethics Committee of China Medical University Hospital (IRB number: CMUH110-REC2–063), and informed consent was waived.

### Study population

We identified a cohort of patients who were initially diagnosed with clinical stage III primary breast cancer in the Taiwan Cancer Registry (TCR) using International Classification of Diseases codes (ICD-9-CM code: 174.9, ICD-10-CM code: C500-C509) and stratified them based on comorbidities and tumor risk factors. A total of 10,896 patients were selected. All the patients received standard treatment as recommended by the American Joint Committee on Cancer (AJCC) 7th edition guidance at the healthcare institute where they were diagnosed between 2010 and 2017. The eligible patients were divided into two groups: one group received inhaled anesthesia during surgery, and the other received propofol-based intravenous anesthesia. The inhaled anesthesia maintenance group was defined as receiving general anesthesia with less than 200mg propofol. The intravenous anesthesia group was defined as receiving total intravenous general anesthesia or general anesthesia with more than 200mg of propofol. All of the patient cohorts were followed up for at least 2 years in the database, with the last follow-up date set on December 31, 2019. We excluded patients with a history of previous malignancy, patients with double cancer, and patients aged younger than 20 years.

### Outcome

In our study, we identified two research outcomes. The primary outcome was the mortality rate, emphasizing the overall mortality rate, as well as the 3-year and 5-year mortality rates. The overall mortality rate was calculated by dividing the number of deaths by every 1000 person-years in the at-risk population during the specified follow-up period. Our secondary outcome pertained to the overall recurrence rate, which was determined by dividing the number of recurrences by every 1000 person-years in the same high-risk population. Of note, the secondary endpoint may not be accurate due to the limitations of the database. We have discussed these limitations in the discussion section. Overall, our methodology facilitated a thorough assessment of both mortality and tumor recurrence as pivotal study endpoints.

### Statistical analysis

Demographic characteristics of patients with stage III breast cancer in the inhaled anesthetic and intravenous anesthetic groups were compared using t-tests for continuous variables and chi-square tests for categorical variables. Multivariate Cox proportional hazards regression models were used to derive adjusted hazard ratios (aHRs) and 95% confidence intervals (CIs) in each cohort, while adjusting for age, sex, comorbidities, and medications. The aforementioned factors are confounders that affect survival according to the literature [22, 23]. Propensity score matching was applied to reduce the impact of confounding factors. We generated cumulative mortality curves to describe the mortality rate over time in the intravenous group and inhaled group. All statistical analyses were performed using SAS System for Windows statistical software, version 9.4 (SAS Institute Inc., Cary, NC). The statistical significance criterion was set at a p-value of less than 0.05 for two-sided testing.

## Results

**Fig 2:**
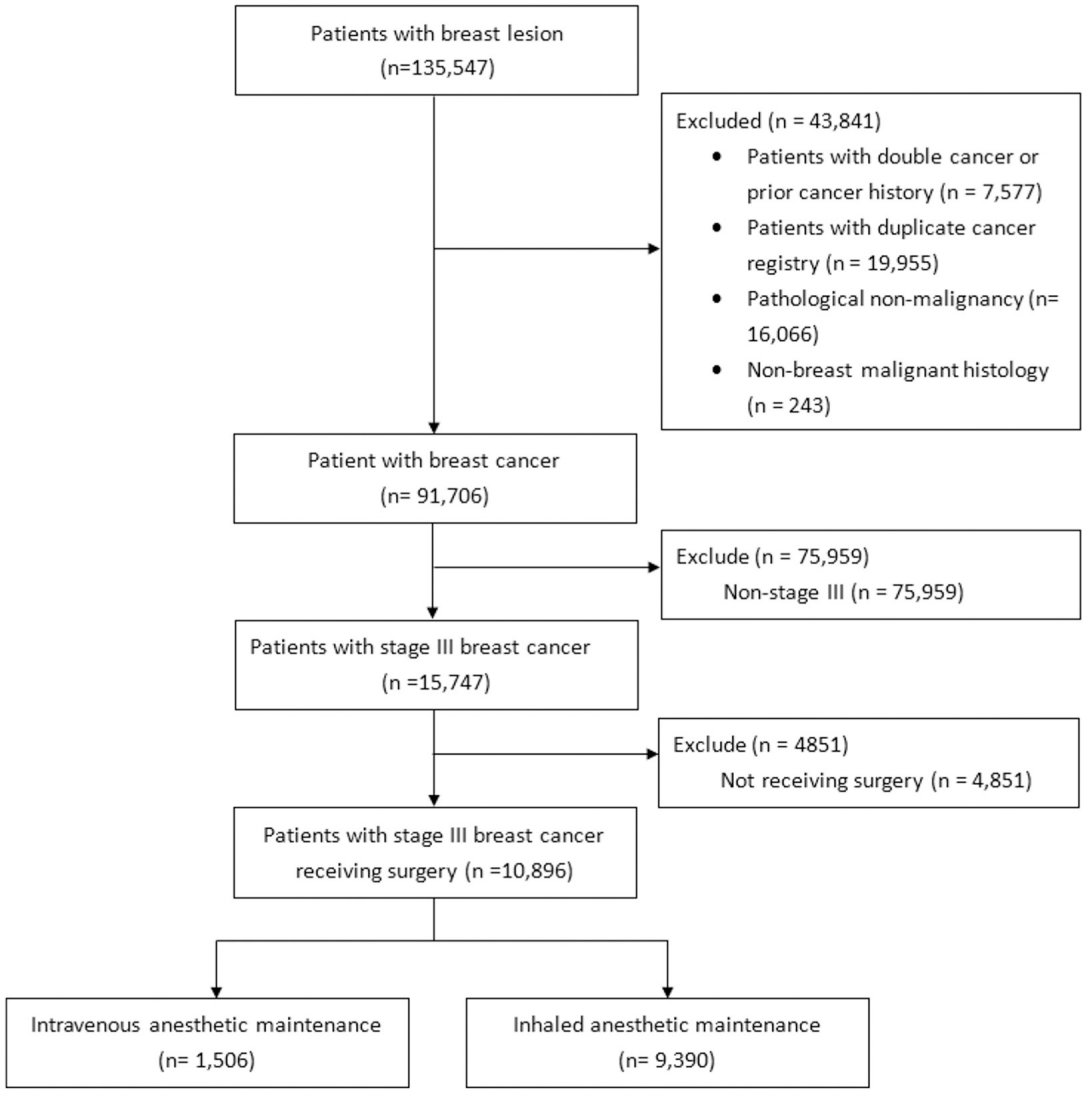
Flowchart of study population. The study analyzed patients with stage III breast cancer who underwent surgical interventions from NHIRD and TCR databases after excluding ineligible cases.

A total of 135,547 patients with breast lesions were identified from a merged database comprising NHIRD and TCR databases. Of these cases, 43,841 were excluded due to double cancer, prior cancer history, pathological non-malignancy, or non-breast malignancy histology. In Fig 2, we identified a cohort of 10,896 patients who had stage III breast cancer and had undergone surgical interventions.

Table 1 showed that of 10,896 patients with stage III breast cancer who received breast cancer surgery and standard treatment in Taiwan, 1,506 received propofol-based intravenous anesthetic maintenance, and 9,390 received inhaled anesthetics maintenance between 2010 and 2017. The mean age of diagnosis was 55.5 ± 12.2 years, and the median follow-up period was 4.71 years (interquartile range, 3.01–7.03) in the total study cohort. The patients were older and had more comorbid conditions, demonstrated as higher Charlson Comorbidity Index (CCI) in the intravenous group before the propensity score matching. Around 4.3% of patients who received more than 200mg of propofol in the inhalation-exposed groups may be caused by inaccurate medical claims or other medical conditions. Both the intravenous group and inhaled group demonstrate similar distribution on body mass index (BMI), CCI, 7th AJCC pathological stage, histological type, histologic grade, subtype, type of surgery, treatment sequence, received standard of care and year of diagnosis after 1:1 randomized propensity-matched. In the propensity-matched cohort, the patients exposed to the inhaled agent group had longer median follow-up time, 6.09 years (interquartile range, 3.96–7.72), compared with the intravenous group, 3.66 years (interquartile range, 2.54–5.36).

**Table 1:** Demographic characteristics of stage III breast cancer patients who received surgery in Taiwan (2010–2017).

Characteristic	Total study cohort, n(%)			p-value	Propensity matched cohort, n(%)			p-value
	Total	IV	IA		Total	IV	IA	
	(n = 10896)	(n = 1506)	(n = 9390)		(n = 2534)	(n = 1267)	(n = 1267)	
Age, year				<0.001				0.02
20–49	3587(32.9)	432(12.0)	3156(88.0)		730(28.8)	381(52.2)	349(47.8)	
50–64	4932(45.3)	633(12.8)	4299(87.2)		1124(44.4)	528(47.0)	596(53.0)	
≥65	2377(21.8)	442(29.4)	1935(20.6)		680(26.8)	358(28.3)	322(25.4)	
Mean (SD)	55.5(12.2)	57.7(13.2)	55.2(12.0)	<0.001	57.1(12.8)	57.2(13.2)	57.0(12.3)	0.65
BMI, Mean (SD)	24.8(4.48)	25.5(4.73)	24.7(4.42)	<0.001	25.4(4.67)	25.4(4.72)	25.4(4.62)	0.88
CCI				<0.001				0.17
0	7652(70.2)	667(44.3)	6985(74.4)		1279(50.5)	632(49.9)	647(51.1)	
1–3	2321(21.3)	493(32.7)	1828(19.5)		808(31.9)	424(33.5)	384(30.3)	
>3	923(8.47)	346(23.0)	577(6.14)		447(17.6)	211(16.7)	236(18.6)	
pT category				<0.001				0.002
T1	2012(18.5)	278(18.5)	1734(18.5)		495(19.5)	238(18.8)	257(20.3)	
T2	5112(46.9)	662(44.0)	4450(47.4)		1184(46.7)	564(44.5)	620(48.9)	
T3	1902(17.5)	285(18.9)	1617(17.2)		435(17.2)	250(19.7)	185(14.6)	
T4	968(8.88)	112(7.44)	856(9.12)		202(7.97)	94(7.42)	108(8.52)	
Unknown	902(8.28)	169(11.2)	733(7.81)		218(8.60)	121(9.55)	97(7.66)	
pN category				<0.001				0.01
N0	1559(14.3)	182(12.1)	1377(14.7)		345(13.6)	152(12.0)	193(15.2)	
N1	1654(15.2)	213(14.1)	1441(15.4)		354(14.0)	181(14.3)	173(13.7)	
N2	4355(40.0)	585(38.8)	3770(40.2)		1015(40.1)	507(40.0)	508(40.1)	
N3	2945(27.0)	423(28.1)	2522(26.9)		718(28.3)	362(28.6)	356(28.1)	
Unknown	383(3.52)	103(6.84)	280(2.98)		102(4.03)	65(5.13)	37(2.92)	
Histology				0.11				0.69
IDC	9379(86.1)	1283(85.2)	8096(86.2)		2167(85.5)	1081(85.3)	1086(85.7)	
ILC	606(5.56)	101(6.71)	505(5.38)		162(6.39)	86(6.79)	76(6.00)	
Others	911(8.36)	122(8.10)	789(8.40)		205(8.09)	100(7.89)	105(8.29)	
Grade				0.89				0.08
Gr.1	622(5.71)	81(5.38)	541(5.76)		140(5.52)	58(4.58)	82(6.47)	
Gr.2	5090(46.7)	717(47.6)	4373(46.6)		1199(47.3)	609(48.1)	590(46.6)	
Gr.3	4142(38.0)	563(37.4)	3579(38.1)		978(38.6)	481(38.0)	497(39.2)	
Unknown	1042(9.56)	145(9.63)	897(9.55)		217(8.56)	119(9.39)	98(7.73)	
LVSI								
Positive	2563(23.5)	360(23.9)	2203(23.5)	<0.001	743(29.3)	323(25.5)	420(33.2)	0.001
Negative	5451(50.0)	857(56.9)	4594(48.9)		1364(53.8)	771(60.9)	593(46.8)	
Unknown	2882(26.5)	289(19.2)	2593(27.6)		427(16.9)	173(13.7)	254(20.1)	
Subtype								
Luminal	6621(60.8)	976(64.8)	5645(60.1)	<0.001	1706(67.3)	852(67.3)	854(67.4)	0.93
HER2 enrich	1234(11.3)	195(13.0)	1039(11.1)	0.03	366(14.4)	179(14.1)	187(14.8)	0.65
Basal	965(8.86)	140(9.30)	825(8.79)	0.52	242(9.55)	125(9.87)	117(9.23)	0.59
Unknown	2076(19.1)	195(13.0)	1881(20.0)		220(8.68)	111(8.76)	109(8.60)	0.89
Type of surgery				<0.001				0.07
BCS	2104(19.3)	275(18.3)	1829(19.5)		472(18.6)	248(19.6)	224(17.7)	
Mastectomy	7574(69.5)	990(65.7)	6584(70.1)		1775(70.1)	862(68.0)	913(72.1)	
Unknown	1218(11.2)	241(16.0)	977(10.4)		287(11.3)	157(12.4)	130(10.3)	
Year of diagnosis				<0.001				0.91
2010–2012	4324(39.7)	914(60.7)	3410(36.3)		1437(56.7)	723(57.1)	714(56.4)	
2013–2015	3867(35.5)	480(31.9)	3387(36.1)		888(35.0)	442(34.9)	446(35.2)	
2016–2018	2705(24.8)	112(7.44)	2593(27.6)		209(8.25)	102(8.05)	107(8.45)	
Median follow-up (IQR)	4.71 (3.01–7.03)	3.60 (2.53–5.44)	4.94 (3.12–7.23)	<0.001	4.64 (2.89–7.07)	3.66 (2.54–5.36)	6.09 (3.96–7.72)	0.001

Abbreviation: IV, intravenous anesthetic group; IA, inhaled anesthetic group; BMI, body mass index; CCI, Charlson Comorbidity Index; IDC, invasive ductal carcinoma; ILC, invasive lobular carcinoma; LVSI, lymph-vascular space invasion; HER2, human epidermal growth factor receptor 2

Before propensity score matching, the overall mortality rate for stage III breast cancer patients who received maintenance with intravenous anesthetics is 6.39%, while the rate for those with inhaled anesthetic maintenance is 4.38%. After adjusting for age, sex, CCI, and medications, the patients who received inhaled anesthetics maintenance remained a lower overall mortality rate than those who received IV (Table 2, adjusted hazard ratio = 0.82, 95% CI: 0.72–0.93). We also found that patients aged above 50 at diagnosis, with comorbidities, normal BMI, 7th AJCC anatomic pathological stage IIIC, invasive ductal carcinoma, higher histologic grade, HER2-enriched subtype, and patients who received mastectomy were statistically significantly associated with a lower overall mortality rate, regardless of the anesthetic techniques. Furthermore, a subgroup analysis comparing IA to non-IA based on age revealed that the overall mortality rate was lower and statistically significant in the IA group for patients aged over 50 years.

**Table 2:** Comparison of overall mortality rate in stage III breast cancer patients considering different baseline characteristics, after adjusting for age, sex, comorbidities, and medications before propensity score matching.

	Non-IA			IA			Univariate		Multivariate	
Variable	Event	Person-Year	IR	Event	Person-Year	IR	HR (95% CI)	P-value	HR (95% CI)	P-value
All	390	6107	63.9	2141	48901	43.8	0.68(0.61, 0.76)	<0.001	0.82(0.72, 0.93)	0.002
Age, year										
20–49	72	1866	38.6	546	17258	31.6	0.82(0.64, 1.05)	0.11	0.94(0.71, 1.26)	0.69
50–64	149	2673	55.7	925	22784	40.6	0.72(0.61, 0.86)	<0.001	0.78(0.64, 0.95)	0.02
>64	169	1568	107.8	670	8859	75.6	0.69(0.58, 0.82)	<0.001	0.82(0.68, 0.99)	0.04
CCI										
0	133	2971	44.8	1531	38259	40.0	0.89(0.75, 1.07)	0.21	0.90(0.74, 1.09)	0.26
1–3	133	1919	69.3	435	8364	52.0	0.75(0.61, 0.91)	0.003	0.76(0.61, 0.95)	0.02
>3	124	1217	101.9	175	2278	76.8	0.75(0.60, 0.94)	0.01	0.76(0.58, 0.99)	0.04
BMI										
<18	77	798	96.5	581	11968	48.6	0.52(0.41, 0.66)	<0.001	0.91(0.43, 1.92)	0.81
18–24	122	2017	60.5	710	17480	40.6	0.68(0.56, 0.82)	<0.001	0.76(0.63, 0.93)	0.01
>24	191	3292	58.0	850	19453	43.7	0.74(0.63, 0.86)	<0.001	0.85(0.72, 1.00)	0.49
pStage										
IIIA	116	2898	40.0	692	22389	30.9	0.76(0.62, 0.93)	0.007	0.93(0.75, 1.16)	0.54
IIIB	24	327	73.5	205	3321	61.7	0.84(0.55, 1.28)	0.40	1.20(0.73, 1.98)	0.47
IIIC	137	1631	84.0	802	12548	63.9	0.76(0.63, 0.91)	0.003	0.78(0.64, 0.96)	0.02
Unknown	113	1251	90.3	442	10643	41.5	0.47(0.38, 0.58)	<0.001	0.72(0.56, 0.93)	0.01
Histology										
IDC	326	5200	62.7	1824	42431	43.0	0.68(0.61, 0.77)	<0.001	0.83(0.72, 0.95)	0.005
ILC	27	427	63.2	120	2560	46.9	0.73(0.48, 1.12)	0.15	0.95(0.59, 1.54)	0.83
Others	37	480	77.0	197	3911	50.4	0.67(0.47, 0.95)	0.03	0.73(0.48, 1.11)	0.15
Grade										
Gr.1	18	353	51.0	78	3080	25.3	0.49(0.29, 0.82)	0.006	0.73(0.36, 1.46)	0.37
Gr.2	144	3028	47.6	861	23310	36.9	0.75(0.63, 0.90)	0.002	0.94(0.76, 1.15)	0.53
Gr.3	178	2213	80.4	979	18218	53.7	0.68(0.58, 0.80)	<0.001	0.81(0.67, 0.97)	0.02
Unknown	50	513	97.5	222	4290	51.7	0.52(0.39, 0.71)	<0.001	0.63(0.44, 0.90)	0.01
LVSI										
Negative	202	3319	60.9	956	20754	46.1	0.85(0.65, 1.12)	0.25	1.07(0.79, 1.44)	0.67
Positive	127	1252	101.5	820	17353	47.3	0.75(0.64, 0.87)	<0.001	0.84(0.72, 0.99)	0.04
Unknown	61	1536	39.7	365	10795	33.8	0.47(0.39, 0.57)	<0.001	0.66(0.51, 0.86)	0.002
Subtype										
Luminal	189	4052	46.7	976	27669	35.3	0.73(0.62, 0.85)	<0.001	0.86(0.73, 1.02)	0.09
HER2 enrich	58	731	79.4	238	5121	46.5	0.59(0.44, 0.79)	<0.001	0.70(0.51, 0.96)	0.03
Basal	58	483	120.2	328	3304	99.3	0.88(0.67, 1.17)	0.38	1.03(0.76, 1.39)	0.86
Unknown	85	842	101	599	12806	46.8	0.47(0.37, 0.59)	<0.001	0.56(0.39, 0.80)	0.002
Type of surgery										
BCS	269	4015	67	284	9490	29.9	1.07(0.74, 1.54)	0.72	1.04(0.70, 1.54)	0.86
Mastectomy	89	909	97.9	1582	34595	45.7	0.68(0.60, 0.77)	<0.001	0.83(0.71, 0.96)	0.01
Unknown	32	1183	27.1	275	4816	57.1	0.61(0.48, 0.78)	<0.001	0.79(0.58, 1.08)	0.15

^1^ Abbreviation: HR, hazard ratio; CI, confidence interval; IR, incidence rate, also indicating mortality rate, per 1000 person-years; IV, intravenous anesthetic group; IA, inhaled anesthetic group; BMI, body mass index; CCI, Charlson Comorbidity Index; IDC, invasive ductal carcinoma; ILC, invasive lobular carcinoma; LVSI, lymph-vascular space invasion; HER2, human epidermal growth factor receptor 2

^2^ Adjusted HR: adjusted for age, sex, comorbidities and medications in Cox proportional hazards regression

We conducted a sensitivity analysis to assess the robustness of our hypothesis. Given the marked discrepancy in cohort sizes, with the intravenous (IV) cohort being significantly smaller than the inhaled cohort, we employed a 1:1 randomized propensity-matched approach to equalize the two groups. The mortality rates per 1,000 person-years were 57.9% for the IV cohort and 47.2% for the inhaled cohort, thereby reinforcing the validity of our empirical findings. After adjusting for potential confounders that could affect survival outcomes, the inhaled anesthetic cohort consistently showed a substantially lower overall mortality rate. This is supported by an adjusted hazard ratio (aHR) of 0.83 (95% CI: 0.71–0.98) compared to the IV cohort, as detailed in Table 3.

**Fig 3:**
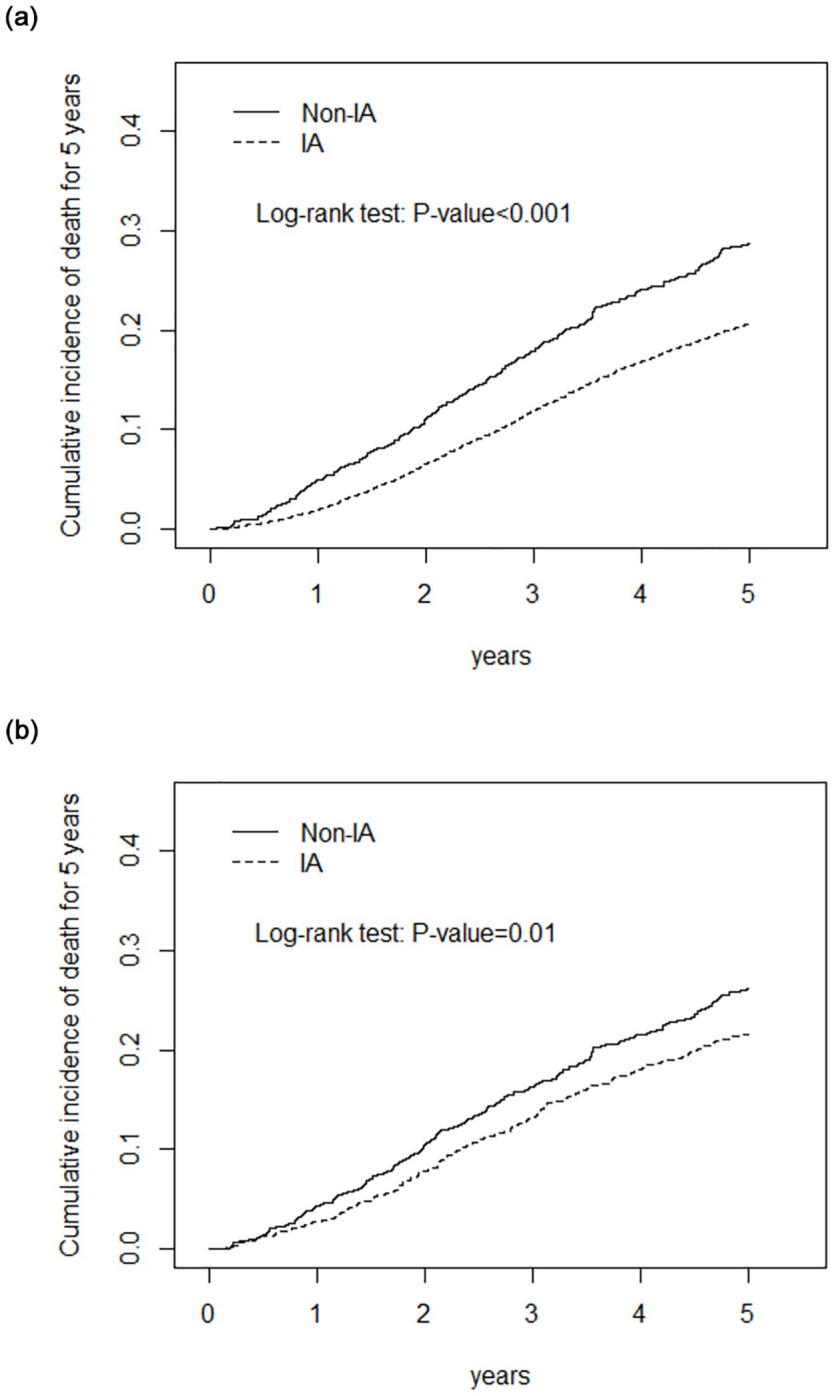
The cumulative mortality rate among stage III breast cancer patients is lower with inhaled anesthetics (IA) compared to non-inhaled anesthetics (non-IA). (A) Before propensity score matching; (B) After propensity score matching.

The cumulative mortality rate across the overall population is depicted in Fig 3. At each time point, the IV group’s cumulative mortality rate consistently exceeded that of the inhaled group (*p* < 0.001). In the IV group, the mortality rate was 16.25% at 3 years and 26.17% at 5 years. In contrast, in the inhaled anesthesia (IA) group, the 3-year mortality rate was 13.2%, and the 5-year rate was 21.59% after propensity score matching. These findings indicate that stage III breast cancer patients who received inhaled anesthetics during surgery had better overall survival over time.

In Table 3, the inhaled cohort exhibited a higher recurrence rate, with an adjusted Hazard Ratio (aHR) for recurrence of 1.28 (95% Confidence Interval [CI]: 1.04–1.58) compared to the cohort receiving intravenous (IV) treatment. This disparity may be attributable to data limitations.

**Table 3:** Comparisons of mortality and recurrence rate between stage III breast cancer patients after propensity score matching.

	Non-IA (N = 1267)			IA (N = 1267)			Univariate		Multivariate	
	Event	Person-Year	IR	Event	Person-Year	IR	HR (95% CI)	P-value	HR (95% CI)	P-value
Recurrence	153	4810	31.8	217	6731	32.2	1.26(1.02, 1.55)	0.03	1.28(1.04, 1.58)	0.02
Mortality	295	5093	57.9	341	7226	47.2	0.83(0.71, 0.97)	0.02	0.83(0.71, 0.98)	0.02

Abbreviation: HR, hazard ratio; CI, confidence interval; IR, incidence rate, also indicating mortality rate, per 1000 person-years; IV, intravenous anesthetic group; IA, inhaled anesthetic group; Adjusted HR: adjusted for age, sex, comorbidities and medications in Cox proportional hazards regression.

## Discussion

This population-based propensity score matching study demonstrates a statistically significant reduction in the mortality rate among patients who received inhaled general anesthesia maintenance compared to propofol-based IV anesthetics in clinical stage III breast cancer. The results remained unchanged even after adjusting for age, gender, comorbidity, and medications. The sensitivity analysis further confirmed the robustness of our findings.

The literature on the impact of anesthetic techniques on advanced cancer stages is scant; nonetheless, our research indirectly suggests that the influence of anesthetic agents on breast cancer may differ across the disease’s various stages. The immune system’s capability to counter tumor progression involves the identification and elimination of cancer cells with mutational changes [24]. According to the established model, breast cancer immunoediting consists of three phases: elimination, equilibrium, and escape, each characterized by distinct immunological responses [25–27]. Anesthetic techniques may have different interactions with the immune system throughout these immunoediting phases, which warrants further investigation to tailor anesthetic strategies that consider the cancer’s immunological profile at each stage.

Research has addressed how volatile anesthetics can modulate cancer signaling pathways [8, 28–30]. For example, anesthetics can impact cancer cell migration and invasion by modulating miRNA and MMP activity, which are pivotal to EMT processes [8, 28, 29, 31, 32]. Wu et al. have shown that Sevoflurane suppresses EMT in breast cancer cells by regulating miR-139–5p/ARF6, while Liu et al. demonstrated that a standard clinical concentration of Sevoflurane inhibits breast cancer cell proliferation through the upregulation of microRNA-203 [8, 30]. These findings align with our results, suggesting a potential protective effect of volatile anesthetics on overall survival in breast cancer patients.

Contrary to our findings, several meta-analyses and randomized controlled trials have shown no significant effects of anesthetics on recurrence-free survival (RFS) and overall survival (OS) in breast cancer, which included a mix of early-stage and advanced-stage patients [25, 33, 34]. Considering that the 5-year overall survival rates for various stages of breast cancer differ substantially, it is plausible that the duration of follow-up in previous studies may not have been adequate to discern long-term effects [35]. Our results, highlighting a lower overall mortality rate in patients receiving inhaled anesthesia, suggest a stage-specific protective effect that merits further investigation.

Moreover, our study indicates a particularly favorable survival outcome for patients over 50 with stage III breast cancer undergoing inhalation anesthesia. Aging is associated with reduced acute inflammatory responses, likely due to immunosenescence and changes in cytokine profiles [36]. Inhalation anesthetics are known to attenuate perioperative inflammatory responses, potentially through the modulation of inflammatory pathways [37]. The combination of age-related and anesthesia-induced reductions in inflammation may synergistically hinder oncogenic progression and lessen postoperative complications. Given the established role of persistent inflammation in tumor initiation, development, and metastasis, mitigating such inflammation could theoretically slow tumor growth and metastatic spread, thereby improving survival rates [38].

While these findings are preliminary, they highlight the importance of considering patient age and inflammatory status in anesthetic planning for oncologic surgery. It is crucial to further investigate these observations to understand the underlying mechanisms and to explore the strategic use of inhalation anesthetics to potentially improve cancer-related outcomes.

In our analysis, we observed an intriguing paradox: the IA group exhibited a higher overall recurrence rate yet a lower overall mortality rate. We attribute this phenomenon to varying data accuracy levels. The data accuracy for the overall mortality rate is high, but that for the overall recurrence rate is low. This discrepancy is due to the constraints imposed by the Taiwan Cancer Registry, which does not mandate institutions to report recurrence data. Although institutions may log any recurrence events occurring after the initial diagnosis, they typically do so within 1 year of the diagnosis, which may be too short a period to document all recurrence events adequately. Moreover, our dataset lacked the comprehensive granularity commonly seen in clinical trials; for instance, we do not have data on progression-free survival (PFS) rates. Consequently, we chose to use the overall recurrence rate as the secondary endpoint. Moreover, we can’t have 3-year and 5-year recurrence rates as secondary endpoints since data logging is usually confined to the first year after diagnosis. In contrast, our mortality data, obtained from national death records in the NHIRD, are both accurate and reliable. Given these considerations, we decided to use the overall mortality rate as the primary endpoint. Further studies are needed to validate secondary endpoints, such as progression-free survival and recurrence-free survival, to ensure their accuracy and reliability. The secondary endpoint results from this study are only for reference.

This study possesses inherent limitations characteristic of retrospective designs. Our reliance on a medical claims database posed challenges, as such databases often lack the granularity required to detail specifics such as drug dosages, duration of anesthesia, and the precise choice of volatile anesthetics. This is a known limitation of many claims databases, where the primary purpose is billing rather than clinical documentation. Consequently, the data may not fully reflect the clinical scenario, carrying a risk of misclassification or omission of pertinent clinical details. Additionally, it must be acknowledged that stage III breast cancer encompasses sub-stages IIIA, IIIB, and IIIC, each with its own inherent heterogeneity. Due to data constraints and the limitations of the statistical methodologies available, separate analyses of each subcategory were not feasible. As a result, these subcategories were aggregated for analysis. This pooling approach may have resulted in the loss of detailed information relevant to the nuances of each subcategory, potentially obscuring specific trends and outcomes associated with each distinct subgroup. Given these challenges and limitations, it is evident that future investigations should consider prospective, randomized controlled trials (RCTs). An RCT would provide a more controlled environment to meticulously examine these variables, ensuring superior data accuracy and clinical relevance.

## Conclusion

In conclusion, our study provides compelling evidence that stage III breast cancer patients who received inhaled anesthetics experienced significantly lower overall mortality rates compared to those in the intravenous propofol-based maintenance group. Specifically, patients over the age of 50 who underwent surgery with propofol-based anesthesia maintenance showed a correlation with a reduced mortality rate. These findings highlight the potential impact of anesthesia technique on patient outcomes in breast cancer. However, further clinical investigations are necessary to validate and expand upon these results. This research has the potential to inform and improve treatment strategies for breast cancer patients, ultimately contributing to better patient care and outcomes in the future.

## Supporting information

S1 Fig (TIFF)

S2 Fig (TIFF)

S3 Fig (TIFF)

S4 Fig (ZIP)
