# Supplementary figures and images for: Inhaled anesthesia associated with reduced mortality in patients with stage III breast cancer: A population-based study
Source: PLoS One. 2024 Mar 1;19(3):e0289519. doi: 10.1371/journal.pone.0289519 (PMC10906904; doi:10.1371/journal.pone.0289519)

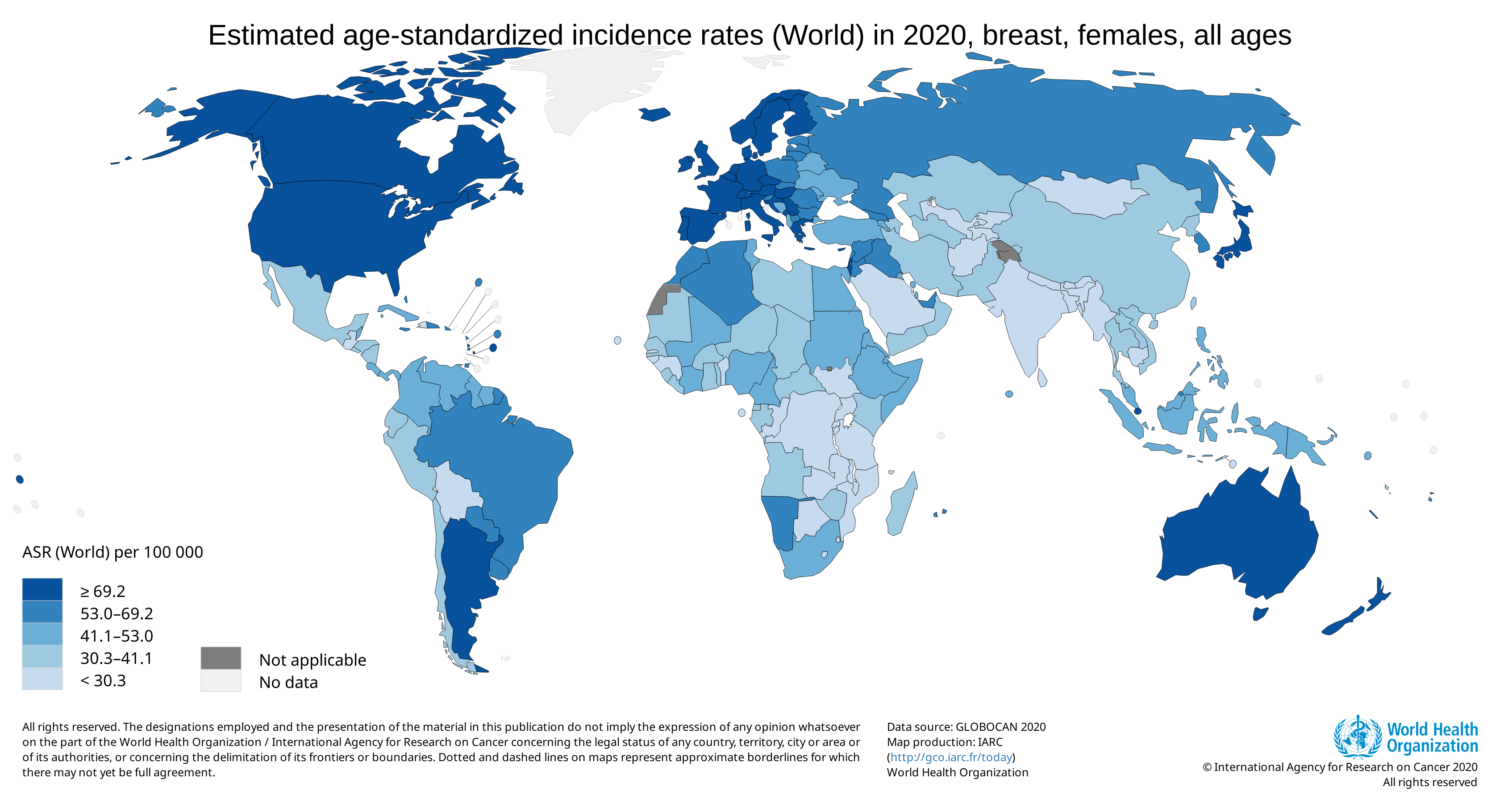

Supplement: S1 Fig — (TIFF) [file pone.0289519.s001.tiff]

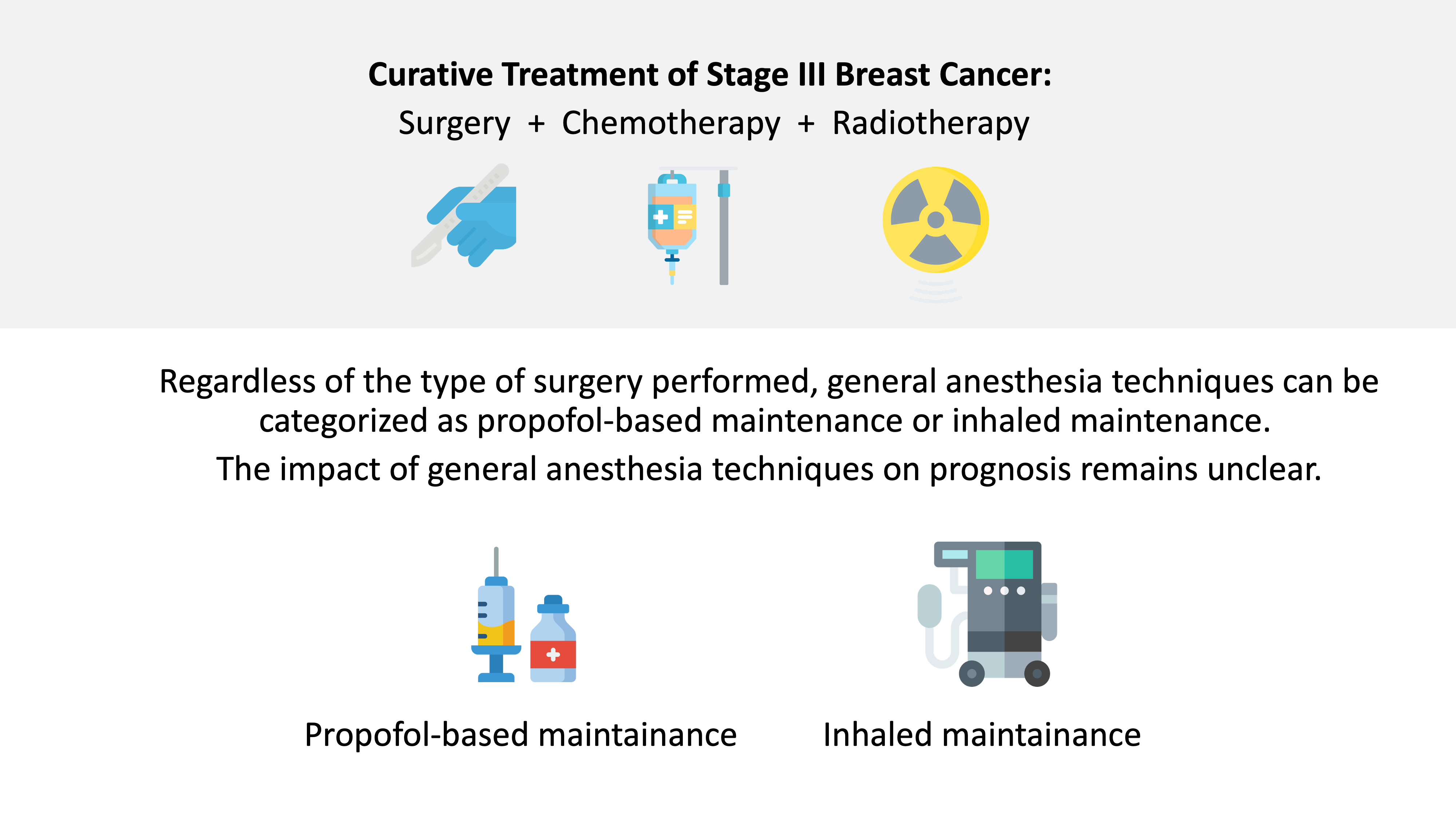

Supplement: S2 Fig — (TIFF) [file pone.0289519.s002.tiff]

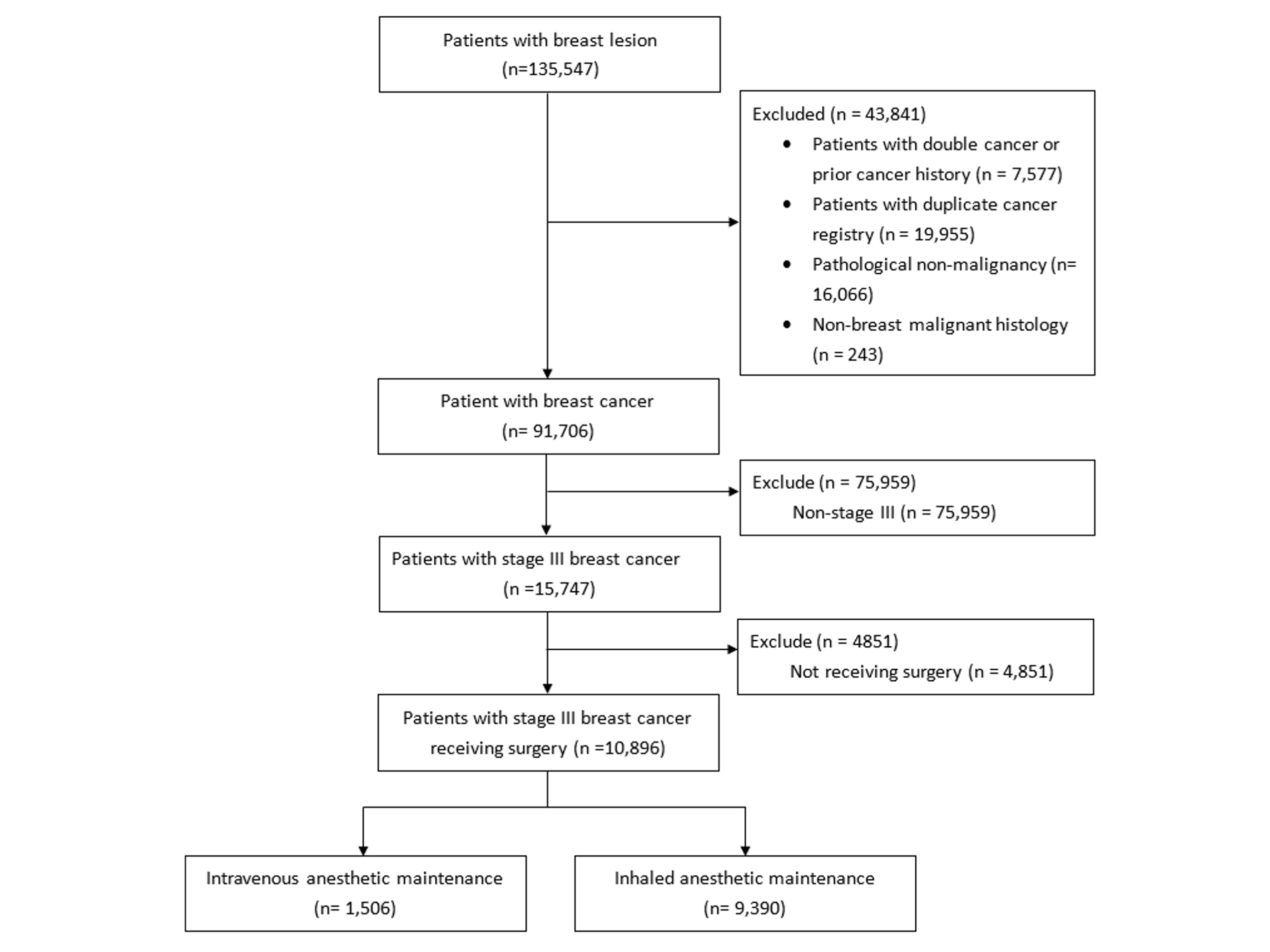

Supplement: S3 Fig — (TIFF) [file pone.0289519.s003.tiff]

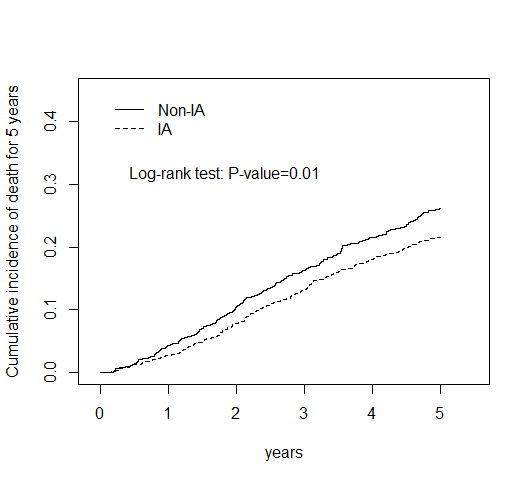

Supplement: S4 Fig — (ZIP) [file pone.0289519.s004.zip › Fig4B.tiff]

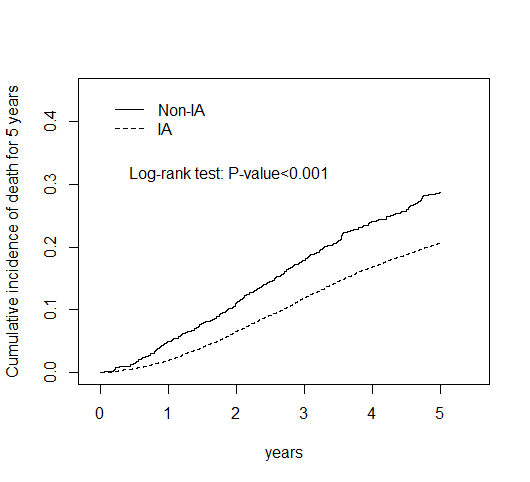

Supplement: S4 Fig — (ZIP) [file pone.0289519.s004.zip › Fig 4A. Rplot_unmatched for die.tiff]
